# CasPEDIA Database: a functional classification system for class 2 CRISPR-Cas enzymes

**DOI:** 10.1093/nar/gkad890

**Published:** 2023-10-27

**Authors:** Benjamin A Adler, Marena I Trinidad, Daniel Bellieny-Rabelo, Elaine Zhang, Hannah M Karp, Petr Skopintsev, Brittney W Thornton, Rachel F Weissman, Peter H Yoon, LinXing Chen, Tomas Hessler, Amy R Eggers, David Colognori, Ron Boger, Erin E Doherty, Connor A Tsuchida, Ryan V Tran, Laura Hofman, Honglue Shi, Kevin M Wasko, Zehan Zhou, Chenglong Xia, Muntathar J Al-Shimary, Jaymin R Patel, Vienna C J X Thomas, Rithu Pattali, Matthew J Kan, Anna Vardapetyan, Alana Yang, Arushi Lahiri, Micaela F Maxwell, Andrew G Murdock, Glenn C Ramit, Hope R Henderson, Roland W Calvert, Rebecca S Bamert, Gavin J Knott, Audrone Lapinaite, Patrick Pausch, Joshua C Cofsky, Erik J Sontheimer, Blake Wiedenheft, Peter C Fineran, Stan J J Brouns, Dipali G Sashital, Brian C Thomas, Christopher T Brown, Daniela S A Goltsman, Rodolphe Barrangou, Virginius Siksnys, Jillian F Banfield, David F Savage, Jennifer A Doudna

**Affiliations:** Innovative Genomics Institute, University of California, Berkeley, CA 94720, USA; California Institute for Quantitative Biosciences (QB3), University of California, Berkeley, CA 94720, USA; Innovative Genomics Institute, University of California, Berkeley, CA 94720, USA; Howard Hughes Medical Institute, University of California, Berkeley, CA 94720, USA; Innovative Genomics Institute, University of California, Berkeley, CA 94720, USA; California Institute for Quantitative Biosciences (QB3), University of California, Berkeley, CA 94720, USA; Innovative Genomics Institute, University of California, Berkeley, CA 94720, USA; Howard Hughes Medical Institute, University of California, Berkeley, CA 94720, USA; Innovative Genomics Institute, University of California, Berkeley, CA 94720, USA; Department of Chemistry, University of California, Berkeley, CA 94720, USA; Innovative Genomics Institute, University of California, Berkeley, CA 94720, USA; California Institute for Quantitative Biosciences (QB3), University of California, Berkeley, CA 94720, USA; Innovative Genomics Institute, University of California, Berkeley, CA 94720, USA; Department of Molecular and Cell Biology, University of California, Berkeley, CA 94720, USA; Innovative Genomics Institute, University of California, Berkeley, CA 94720, USA; Department of Molecular and Cell Biology, University of California, Berkeley, CA 94720, USA; Innovative Genomics Institute, University of California, Berkeley, CA 94720, USA; Department of Molecular and Cell Biology, University of California, Berkeley, CA 94720, USA; Innovative Genomics Institute, University of California, Berkeley, CA 94720, USA; Department of Earth and Planetary Sciences, University of California, Berkeley, CA 94720, USA; Innovative Genomics Institute, University of California, Berkeley, CA 94720, USA; Department of Earth and Planetary Sciences, University of California, Berkeley, CA 94720, USA; Department of Environmental Science, Policy, and Management, University of California, Berkeley, CA 94720, USA; EGSB Division, Lawrence Berkeley National Laboratory, Berkeley, CA 94720, USA; Innovative Genomics Institute, University of California, Berkeley, CA 94720, USA; Department of Molecular and Cell Biology, University of California, Berkeley, CA 94720, USA; Innovative Genomics Institute, University of California, Berkeley, CA 94720, USA; Department of Molecular and Cell Biology, University of California, Berkeley, CA 94720, USA; Innovative Genomics Institute, University of California, Berkeley, CA 94720, USA; California Institute for Quantitative Biosciences (QB3), University of California, Berkeley, CA 94720, USA; Innovative Genomics Institute, University of California, Berkeley, CA 94720, USA; California Institute for Quantitative Biosciences (QB3), University of California, Berkeley, CA 94720, USA; Innovative Genomics Institute, University of California, Berkeley, CA 94720, USA; University of California, Berkeley - University of California, San Francisco Graduate Program in Bioengineering, University of California, Berkeley, Berkeley, CA 94720, USA; Department of Chemistry, University of California, Berkeley, CA 94720, USA; Innovative Genomics Institute, University of California, Berkeley, CA 94720, USA; California Institute for Quantitative Biosciences (QB3), University of California, Berkeley, CA 94720, USA; Graduate School of Life Sciences, Utrecht University, 3584 CS Utrecht, UT, The Netherlands; Innovative Genomics Institute, University of California, Berkeley, CA 94720, USA; Howard Hughes Medical Institute, University of California, Berkeley, CA 94720, USA; Innovative Genomics Institute, University of California, Berkeley, CA 94720, USA; Department of Molecular and Cell Biology, University of California, Berkeley, CA 94720, USA; Innovative Genomics Institute, University of California, Berkeley, CA 94720, USA; Department of Molecular and Cell Biology, University of California, Berkeley, CA 94720, USA; Innovative Genomics Institute, University of California, Berkeley, CA 94720, USA; California Institute for Quantitative Biosciences (QB3), University of California, Berkeley, CA 94720, USA; Innovative Genomics Institute, University of California, Berkeley, CA 94720, USA; Department of Molecular and Cell Biology, University of California, Berkeley, CA 94720, USA; Innovative Genomics Institute, University of California, Berkeley, CA 94720, USA; Innovative Genomics Institute, University of California, Berkeley, CA 94720, USA; Department of Chemistry, University of California, Berkeley, CA 94720, USA; Innovative Genomics Institute, University of California, Berkeley, CA 94720, USA; Department of Molecular and Cell Biology, University of California, Berkeley, CA 94720, USA; Innovative Genomics Institute, University of California, Berkeley, CA 94720, USA; Department of Pediatrics, Division of Allergy, Immunology, and Bone Marrow Transplantation, University of California, San Francisco, CA 94158, USA; Innovative Genomics Institute, University of California, Berkeley, CA 94720, USA; Innovative Genomics Institute, University of California, Berkeley, CA 94720, USA; Department of Molecular and Cell Biology, University of California, Berkeley, CA 94720, USA; Department of Molecular and Cell Biology, University of California, Berkeley, CA 94720, USA; Department of Chemistry and Biochemistry, Hampton University, Hampton, VA 23668, USA; Innovative Genomics Institute, University of California, Berkeley, CA 94720, USA; Innovative Genomics Institute, University of California, Berkeley, CA 94720, USA; Innovative Genomics Institute, University of California, Berkeley, CA 94720, USA; Monash Biomedicine Discovery Institute, Department of Biochemistry and Molecular Biology, Faculty of Medicine, Nursing and Health Sciences, Monash University, Clayton, VIC 3168, Australia; Monash Biomedicine Discovery Institute, Department of Biochemistry and Molecular Biology, Faculty of Medicine, Nursing and Health Sciences, Monash University, Clayton, VIC 3168, Australia; Monash Biomedicine Discovery Institute, Department of Biochemistry and Molecular Biology, Faculty of Medicine, Nursing and Health Sciences, Monash University, Clayton, VIC 3168, Australia; School of Molecular Sciences, Arizona State University, Tempe, AZ 85281, USA; Arizona State University-Banner Neurodegenerative Disease Research Center at the Biodesign Institute, Arizona State University, Tempe, AZ 85281, USA; Center for Molecular Design and Biomimetics, The Biodesign Institute, Arizona State University, Tempe, AZ 85281, USA; LSC-EMBL Partnership Institute for Genome Editing Technologies, Life Sciences Center, Vilnius University, Vilnius 10257, Lithuania; Department of Biological Chemistry and Molecular Pharmacology, Harvard Medical School, Boston, MA 02115, USA; RNA Therapeutics Institute, University of Massachusetts Chan Medical School, Worcester, MA 01655, USA; Program in Molecular Medicine, University of Massachusetts Chan Medical School, Worcester, MA 01655, USA; Li Weibo Institute for Rare Diseases Research, University of Massachusetts Chan Medical School, Worcester, MA 01655, USA; Department of Microbiology and Cell Biology, Montana State University, Bozeman, MT 59717, USA; Department of Microbiology and Immunology, University of Otago, Dunedin 9016, New Zealand; Genetics Otago, University of Otago, Dunedin 9016, New Zealand; Bioprotection Aotearoa, University of Otago, Dunedin 9016, New Zealand; Maurice Wilkins Centre for Molecular Biodiscovery, University of Otago, Dunedin 9016, New Zealand; Department of Bionanoscience, Delft University of Technology, 2629 HZ Delft, Netherlands; Kavli Institute of Nanoscience, 2629 HZ Delft, The Netherlands; Roy J. Carver Department of Biochemistry, Biophysics and Molecular Biology, Iowa State University, Ames, IA 50011, USA; Metagenomi, Inc., Emeryville, CA 94608, USA; Metagenomi, Inc., Emeryville, CA 94608, USA; Metagenomi, Inc., Emeryville, CA 94608, USA; Innovative Genomics Institute, University of California, Berkeley, CA 94720, USA; Department of Food, Bioprocessing and Nutrition Sciences, North Carolina State University, Raleigh, NC 27606, USA; Institute of Biotechnology, Life Sciences Center, Vilnius University, Vilnius 10257, Lithuania; Innovative Genomics Institute, University of California, Berkeley, CA 94720, USA; Department of Earth and Planetary Sciences, University of California, Berkeley, CA 94720, USA; Department of Environmental Science, Policy, and Management, University of California, Berkeley, CA 94720, USA; EGSB Division, Lawrence Berkeley National Laboratory, Berkeley, CA 94720, USA; The University of Melbourne, Parkville, VIC 3052, Australia; Innovative Genomics Institute, University of California, Berkeley, CA 94720, USA; Howard Hughes Medical Institute, University of California, Berkeley, CA 94720, USA; Department of Molecular and Cell Biology, University of California, Berkeley, CA 94720, USA; Innovative Genomics Institute, University of California, Berkeley, CA 94720, USA; California Institute for Quantitative Biosciences (QB3), University of California, Berkeley, CA 94720, USA; Howard Hughes Medical Institute, University of California, Berkeley, CA 94720, USA; Department of Chemistry, University of California, Berkeley, CA 94720, USA; Department of Molecular and Cell Biology, University of California, Berkeley, CA 94720, USA; MBIB Division, Lawrence Berkeley National Laboratory, Berkeley, CA 94720, USA; Gladstone Institutes, University of California, San Francisco, CA 94158, USA

## Abstract

CRISPR-Cas enzymes enable RNA-guided bacterial immunity and are widely used for biotechnological applications including genome editing. In particular, the Class 2 CRISPR-associated enzymes (Cas9, Cas12 and Cas13 families), have been deployed for numerous research, clinical and agricultural applications. However, the immense genetic and biochemical diversity of these proteins in the public domain poses a barrier for researchers seeking to leverage their activities. We present CasPEDIA (http://caspedia.org), the Cas Protein Effector Database of Information and Assessment, a curated encyclopedia that integrates enzymatic classification for hundreds of different Cas enzymes across 27 phylogenetic groups spanning the Cas9, Cas12 and Cas13 families, as well as evolutionarily related IscB and TnpB proteins. All enzymes in CasPEDIA were annotated with a standard workflow based on their primary nuclease activity, target requirements and guide-RNA design constraints. Our functional classification scheme, CasID, is described alongside current phylogenetic classification, allowing users to search related orthologs by enzymatic function and sequence similarity. CasPEDIA is a comprehensive data portal that summarizes and contextualizes enzymatic properties of widely used Cas enzymes, equipping users with valuable resources to foster biotechnological development. CasPEDIA complements phylogenetic Cas nomenclature and enables researchers to leverage the multi-faceted nucleic-acid targeting rules of diverse Class 2 Cas enzymes.

## Graphical Abstract

**Graphical Abstract:**
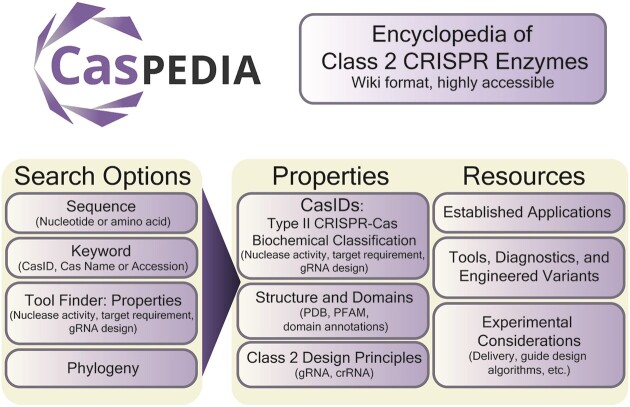

## Introduction

CRISPR-Cas (clustered regularly interspaced short palindromic repeats, CRISPR-associated) systems provide adaptive immunity in bacteria and archaea through RNA-guided recognition and Cas-mediated destruction of foreign nucleic acids (1,2). These immune systems are exceptionally diverse, occurring as 6 types and 33 subtypes in line with recent classification (3). Beginning with the discovery of RNA-guided endonuclease activity conferred by Cas9, insights into the enzymatic activities of CRISPR Cas enzymes have precipitated a veritable wave of biotechnological innovation (4–6).

In particular, the Class 2 Cas enzymes have been a driver of biotechnological development owing to their single-protein nature. Class 2 Cas enzymes can be separated into 3 families: Cas9, Cas12 and Cas13 from Type II, V and VI CRISPR systems respectively (3). Because these proteins all employ a processed CRISPR RNA (crRNA) to guide protein activity towards a sequence of interest, these proteins can all be easily ‘programmed’ to target unique sequences of interest by simple design of a spacer (i.e. targeting) sequence. However, as researchers have explored the genetic diversity of these systems, it has become clear that (i) the RNA-guided biochemical activity, (ii) constraints on targeting context and (iii) ways crRNAs are processed differ dramatically across - and within - families. While these differences reflect opportunities for biotechnological development, there does not yet exist a centralized resource for comparing biochemical activity to complement existing genetic classification efforts (3). It remains difficult for these enzymes to be functionally compared and contrasted across and within subfamilies.

Here, we present CasPEDIA, http://caspedia.org, providing users with summary information about the capabilities and limitations of Class 2 Cas technologies to facilitate tool selection and to highlight opportunities for future biotechnological development. We introduce CasID, a Cas enzyme classification scheme, to facilitate functional comparison between RNA-guided Class 2 Cas enzymes. The optimal selection of a CRISPR enzyme depends heavily on the intended application and CasPEDIA allows for efficient comparison between enzymes by both their biochemical properties and their previously established uses. As a flexible database, CasPEDIA can be updated to accommodate the emergence of novel CRISPR-Cas enzymes and their applications.

## Main features of CasPEDIA

**Figure 1.**
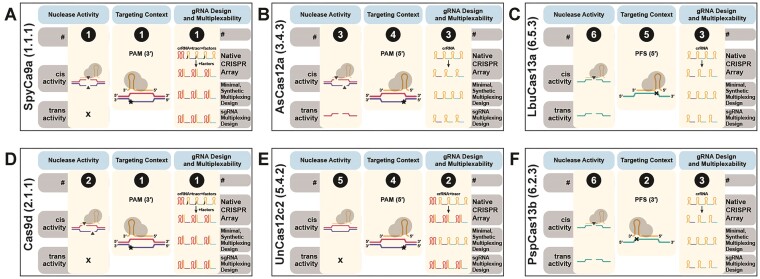
CasID enzymatic labels for biotechnologically important Cas enzymes. Examples are shown for (**A**) SpyCas9a (1.1.1), (**B**) Cas9d (2.1.1), (**C**) AsCas12a (3.4.3), (**D**) UnCas12c2 (5.4.2), (**E**) LbuCas13a (6.5.3), (**F**) PspCas13b (6.2.3). For a complete description and list of CasID values and their definition, please refer to http://caspedia.org/.

CasPEDIA introduces a systematic, enzymatic nomenclature for the functional classification of Class 2 Cas proteins, summarized in Table 1. This classification, termed CasID, is directly inspired by the ENZYME Classification (E.C.) system, but is tailored to the unique properties of these RNA-guided enzymes (7). Each enzyme in CasPEDIA receives a 3-decimal number reflecting its biochemical activities as RNA-guided enzymes. Briefly, CasPEDIA’s classification schema can be seen in Table 1 and is summarized here. Familiar to most CRISPR biotechnologists is ‘Nuclease Activity’, describing which nucleic acids are predominantly cut in *cis* (i.e. guide RNA-targeted) or in *trans* (i.e. non-guide RNA targeted). Additionally, we provide insight into ‘Targeting Context’ or constraints on sequences that neighbor the targeted sequence (e.g. protospacer-adjacent motif (PAM) (required adjacent sequence for targeting) or protospacer-flanking sequence (PFS) (activity-suppressing, adjacent sequence during targeting)). Finally, we provide ‘gRNA Design and Multiplexability'' to enable design of multiplexed guide RNAs (gRNAs) from a native CRISPR locus. For instance, as exhibited in Figure 1A, SpyCas9 can be summarized by CasID 1.1.1, meaning SpyCas9 is an RNA-guided enzyme with targeted double-stranded DNA (dsDNA) activity with blunt cleavage and no *trans*-activity, employs a 3′ PAM positioning, and requires multiple synthetic gRNAs for multiplexable design. The enzymatic classification of Class 2 CRISPR proteins is intended to complement evolutionary classification efforts (3,8). In tandem with phylogenetic classification, we hope that the consolidation of enzymatic and sequence information fosters the further development of CRISPR-based biotechnologies.

**Table 1.** Cas Enzyme Classification. See http://caspedia.org for diagrams

Dimension	Value	Description
**Primary Nuclease Activity**	**1**	**Targets dsDNA + no *trans*-activity. Cleavage products are predominantly blunt**. However, additional trimming of DNA cleavage products may occur on a timescale much slower than that of the initial cuts. RNA-guided RuvC domains are also capable of targeted, PAM-independent ssDNA cleavage.
	**2**	**Targets dsDNA + no *trans*-activity. Staggered cleavage products**. RNA-guided RuvC domains are also capable of targeted, PAM-independent ssDNA cleavage.
	**3**	**Targets dsDNA (or ssDNA) + *trans*-ssDNase activity. Staggered cleavage products**. RNA-guided RuvC domains are also capable of PAM-independent ssDNA cleavage.
	**4**	**Targets dsDNA (primarily nicking) + *trans*-ssDNase activity**. Cleavage products are predominantly nicked on a single strand on short timescales, but these enzymes retain the capacity to create double-strand breaks at a slow rate.
	**5**	**Targets dsDNA (binding only)**
	**6**	**Targets RNA + *trans*-RNase activity**
	**7**	**Targets RNA + *trans*-RNase + *trans*-ssDNase activity**
	**8**	**Targets RNA + *trans*-RNase + *trans*-ssDNase + *trans*-dsDNase activity**
	**-**	**Unknown**
		
**Target Requirement**	**1**	**3′ Protospacer-adjacent motif (PAM)**. This is a required sequence encoded in the non-targeted strand. 3′ positioning also means the 3′ CRISPR repeat is used.
	**2**	**3′ Protospacer-flanking sequence (PFS)**. This is a prohibited sequence encoded in the targeted strand also referred to as an anti-tag. 3′ positioning also means the 3′ CRISPR repeat is used.
	**3**	**3′ No constraints**. 3′ positioning means the 3′ CRISPR repeat is used.
	**4**	**5′ Protospacer-adjacent motif (PAM)**. This is a required sequence encoded in the non-targeted strand. 5′ positioning also means the 5′ CRISPR repeat is used.
	**5**	**5′ Protospacer-flanking sequence (PFS)**. This is a prohibited sequence encoded in the targeted strand also referred to as an anti-tag. 5′ positioning also means the 5′ CRISPR repeat is used.
	**6**	**5′ No constraints**. 5′ positioning means the 5′ CRISPR repeat is used.
	**-**	**Unknown**
		
**Guide RNA (gRNA) design + Multiplexing**	**1**	**crRNA + tracrRNA + non-CRISPR-associated endogenous factors in the native host** required for CRISPR processing
	**2**	**crRNA + tracrRNA** required for CRISPR processing
	**3**	**crRNA** required for CRISPR processing
	**4**	**ωRNA**
	**-**	**Unknown**

The website's Tool Finder (http://caspedia.org/tool_finder.html) may be used to explore and tabulate enzymes that possess each feature below.

**Figure 2.**
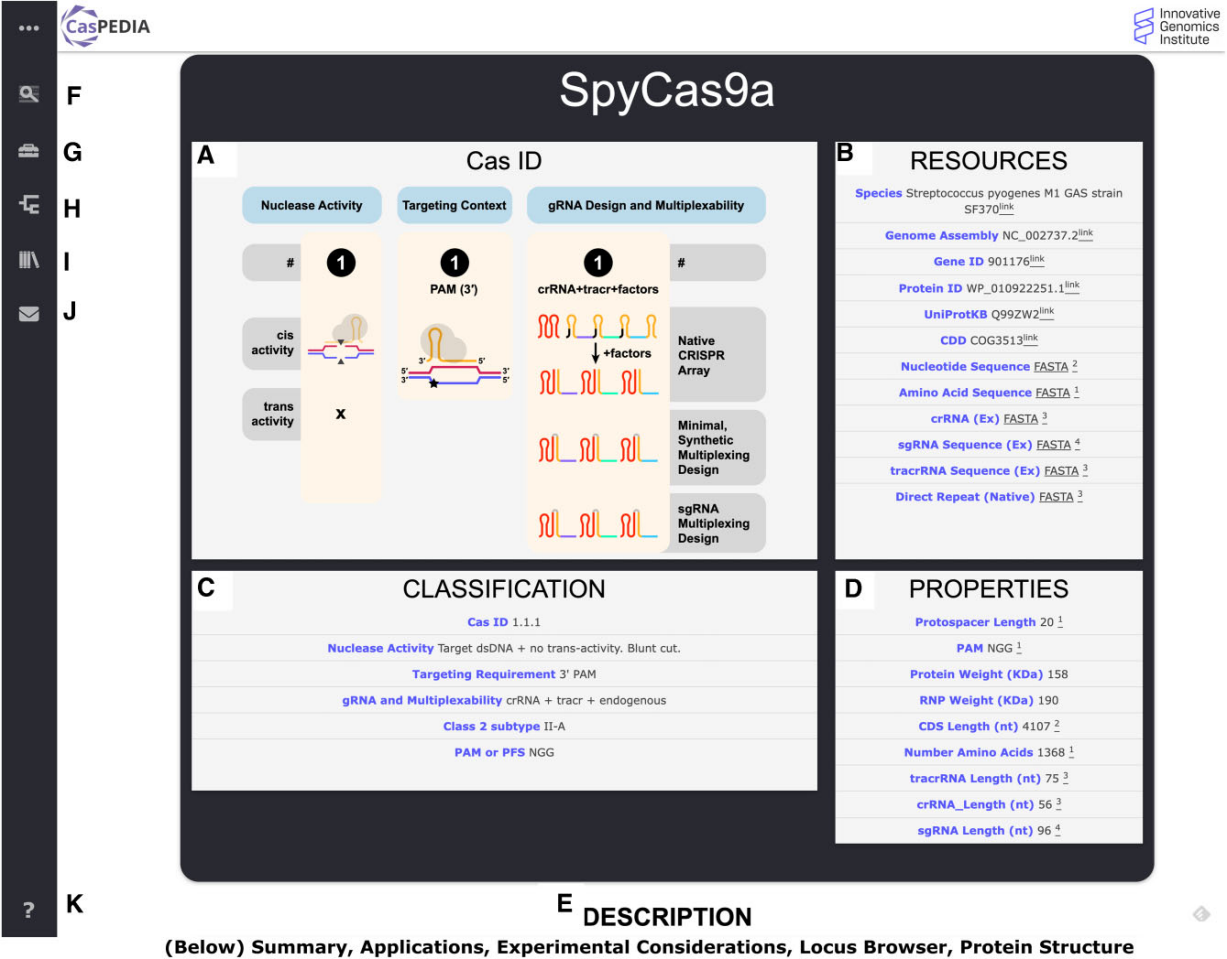
Overview of CasPEDIA Entry for SpyCas9a (1.1.1) from the database. (**A**) CasID diagram and functional description. (**B**) Resources for accessing native sequences and gRNA design for the Cas enzyme. (**C**) Functional and phylogenetic classification of SpyCas9 (CasID 1.1.1). (**D**) Biological properties of this Cas enzyme, including protein, gene and gRNA properties. (**E**) Overview of the Cas enzyme including a summary of the enzyme, applications, experimental considerations, protein structure and gene browser (below the visualized portion). (**F**) Link to homepage containing CasID Definitions and search bar, accommodating queries for Cas enzymes by CasID, protein name or protein family. (**G**) Icon for Tool Finder, where users can search CasPEDIA for enzymes with specific properties. (**H**) Redirects to Cas Phylogeny page for browsing the website by protein family. (**I**) Tool Glossary of common CRISPR-Cas systems. (**J**) Contact Page. (**K**) FAQ and general information.

CasPEDIA is organized in wiki format, with dedicated web pages for an initial set of 33 nucleases. Shown in Figure 2, each wiki contains 7 sections: Quick Review, Summary, Applications, Experimental Considerations, Nucleotide Sequence, and Protein Structure. The Quick Review section, located at the top of the page, enables rapid access to essential information including: enzyme classification (a description of the CasID and phylogenetic classification), core properties (e.g. protospacer length, PAM/PFS, length of the nucleotide coding-sequence, etc.) and external resources (e.g. RefSeq identifiers for the gene and protein, UniProtKB ID, Conserved Domains Database IDs, etc.) (9–11). Next, is a high-level Summary section, detailing the nuclease's origins, novel properties, and common uses. The Applications section then provides a literature review for the enzyme with sub-headers for Gene Editing examples in model organisms, Tools and Diagnostics utilizing the enzyme and Engineered Variants with expanded properties. This is followed by the Experimental Considerations section, a brief introduction to performing experiments with the Cas enzyme. It includes details on construct design, appropriate delivery modalities and a list of algorithms for gRNA design. Nucleotide Sequence is also discussed, complete with downloads and a genome browser, created with igv.js (12), demonstrating the nuclease's sequence and the architecture of its CRISPR array. The subsequent section covers Protein Structure, which includes a summary of the protein's domains from UniProt (11), Pfam annotations (13) and structures from the PDB (14), or predicted with AlphaFold2 (15), visualized using 3Dmol.js (16). Citations are provided for all wiki content and indexed at the bottom of the webpage. To assist users in locating relevant wiki entries, CasPEDIA includes extensive search features and navigational pages, discussed below.

## Navigating CasPEDIA

CasPEDIA provides multiple search tools to connect users with pertinent CRISPR-Cas enzymes and wiki content (Figure 2). Scientists can use the search bar, located on the homepage, to search for Cas nucleases by name (e.g. AsCas12a, SpyCas9a), RefSeq protein ID, or function using CasID nomenclature. Each search returns a table containing matching protein entries and displays for each entry: Enzyme Name, CasID, Protein Accession (RefSeq protein ID, when available), Nuclease Activity, Targeting Requirement, gRNA Design and Multiplexability, and PAM. Similarly, the search bar can also be used to query the database for a protein sequence using DELTA-BLAST (17) with default parameters. This approach allows for remote homology detection with the support of NCBI’s Conserved Domain Database (CDD) (10) for domain-enhanced sequence searches across the CasPEDIA database. The resulting table is sortable by all fields, including E-value, to assist users in finding a nuclease of interest.

A separate page, entitled Tool Finder, directs users through a series of drop-down menus (fields include: Cis-Activity Substrate, Trans-Activity Substrate, Targeting Requirements and gRNA Design and Multiplexability), which generates a table of all Class 2 systems within CasPedia that demonstrate or conservatively predicted to demonstrate the selected properties.

CasPEDIA also supports phylogenetic navigation, complementing evolutionary classifications from previous studies (3,8). The Phylogeny page of the website provides summaries of Type II, Type V and Type VI systems which make up Class 2. We provide dedicated pages for each system type, containing subtype descriptions and an interactive tree whose leaves redirect to wiki entries.

While CasPEDIA wiki entries are organized by protein type (i.e. nuclease name and corresponding species) and CasID, users may also locate information for examples of engineered variants and gene-editing tools. Term searches for engineered variants are unsupported at this time, but variant details can be identified by searching the parental enzyme by name, and scanning the "Engineered Variants" section of the parental wiki entry. Furthermore, a designated page for fusion proteins is available (i.e. Tool Glossary), organizing the expanding list of base editors and prime editors by function, as well as proteins used for CRISPR interference (CRISPRi), CRISPR activation (CRISPRa) and other tools.

## CasPEDIA data curation

CasPEDIA is a community project, curated from the literature by a panel of CRISPR researchers. Wiki content was managed through a series of forms, which were distributed amongst curators and editors for completion. To ensure accuracy and objectivity, citations from peer-reviewed publications and databases were required. Citations are provided at the base of each page. Structural and sequence information were taken from literature or databases like PDB, NCBI, UniProt and Pfam. The CasPEDIA Consortium and Scientific Communications Team at the Innovative Genomics Institute reviewed all entries prior to initial release.

Additionally, we visualized CasPEDIA’s enzymatic classification efforts against the current genetic classification of Class 2 CRISPR systems (3,8). Phylogenetic trees were constructed for each Class 2 Type from comprehensive datasets for Cas9, Cas12 and Cas13 proteins (3,18–21). Trees were constructed with IQ-TREE from MUSCLE aligned sequences, and visualized in iTOL (22–24).

## Future developments

Currently, CasPEDIA only contains entries for the enzymatic activities of Cas effectors in Class 2 CRISPR-Cas systems, as there is limited distinction between the enzymatic activity of the protein and the mature CRISPR-Cas complex. The current CasPEDIA entries include representatives from all 27 phylogenetic subtypes encoded within the Cas9, Cas12 and Cas13 families. We also provide entries including related proteins IscB (HEARO) and TnpB, important variants used in biotechnological applications, and enzymatic subtypes (ex. Cas12c1 versus Cas12c2). Class 2 CRISPR system derived enzymes represent only a fraction of the overall Cas protein diversity (3). Class 1 CRISPR-Cas systems and CRISPR adaptation, comprise the most abundant CRISPR systems and enzymes across bacterial and archaeal genomes (3). Owing to their multi-protein nature, Class 1 CRISPR-Cas interference complexes coordinate multiple enzymatic activities in target nucleic acid recognition and their adoption for biotechnology has thus been difficult (2,25–27). Adaptations of CasID for these enzyme complexes would facilitate greater adoption and subsequent innovation by the biotechnology community and is a clear priority for future iterations. Additionally, new Class 2 CRISPR-Cas systems are emerging at a rapid pace. During the preparation of the CasPEDIA database alone, seven new systems were reported (20,28–34). We anticipate that many new systems will emerge by the next update of CasPEDIA.

CasPEDIA is an actively evolving database, which will grow through community engagement and sustained content management. CRISPR scientists are encouraged to contact the CasPEDIA Consortium to suggest new wiki entries and features, as well as update current wikis with emergent discoveries. These efforts will maintain the relevancy of the database as a useful resource for future scientists. Prospective volunteers can follow detailed directions on the Contact page of the website to contribute.

## Data Availability

CasPEDIA is freely accessible at http://caspedia.org, and data is licensed under Creative Commons Attribution 4.0 International License (CC BY 4.0). The website is compatible with all devices, including tablets and mobile phones. A complete inventory of enzymes in CasPEDIA, along with CasID numbers, can be downloaded on the Tool Finder page. Text content for the wikis is available upon request, with more information provided on the Contact page of the website. Illustrations from CasPEDIA are available for non-commercial use under a Creative Commons Attribution-NonCommercial-ShareAlike 4.0 International License (CC BY-NC-SA 4.0). Please credit “Innovative Genomics Institute, University of California, Berkeley”.

## References

[1] Barrangou R. , Fremaux C., Deveau H., Richards M., Boyaval P., Moineau S., Romero D.A., Horvath P. CRISPR provides acquired resistance against viruses in prokaryotes. Science. 2007; 315:1709–1712. 17379808 10.1126/science.1138140

[2] Brouns S.J.J. , Jore M.M., Lundgren M., Westra E.R., Slijkhuis R.J.H., Snijders A.P.L., Dickman M.J., Makarova K.S., Koonin E.V., van der Oost J. Small CRISPR RNAs guide antiviral defense in prokaryotes. Science. 2008; 321:960–964. 18703739 10.1126/science.1159689 PMC5898235

[3] Makarova K.S. , Wolf Y.I., Iranzo J., Shmakov S.A., Alkhnbashi O.S., Brouns S.J.J., Charpentier E., Cheng D., Haft D.H., Horvath P. et al. Evolutionary classification of CRISPR-Cas systems: a burst of class 2 and derived variants. Nat. Rev. Microbiol. 2020; 18:67–83. 31857715 10.1038/s41579-019-0299-x PMC8905525

[4] Jinek M. , Chylinski K., Fonfara I., Hauer M., Doudna J.A., Charpentier E. A programmable dual-RNA-guided DNA endonuclease in adaptive bacterial immunity. Science. 2012; 337:816–821. 22745249 10.1126/science.1225829 PMC6286148

[5] Gasiunas G. , Barrangou R., Horvath P., Siksnys V. Cas9-crRNA ribonucleoprotein complex mediates specific DNA cleavage for adaptive immunity in bacteria. Proc. Natl. Acad. Sci. U.S.A. 2012; 109:E2579–E2586. 22949671 10.1073/pnas.1208507109 PMC3465414

[6] Wang J.Y. , Doudna J.A. CRISPR technology: a decade of genome editing is only the beginning. Science. 2023; 379:eadd8643. 36656942 10.1126/science.add8643

[7] Bairoch A. The ENZYME database in 2000. Nucleic Acids Res. 2000; 28:304–305. 10592255 10.1093/nar/28.1.304 PMC102465

[8] Koonin E.V. , Gootenberg J.S., Abudayyeh O.O. Discovery of diverse CRISPR-Cas systems and expansion of the genome engineering toolbox. Biochemistry. 2023; 10.1021/acs.biochem.3c00159 PMC10734277 37192099

[9] O’Leary N.A. , Wright M.W., Brister J.R., Ciufo S. Reference sequence (RefSeq) database at NCBI: current status, taxonomic expansion, and functional annotation. Nucleic Acids Res. 2015; 10.1093/nar/gkv1189 PMC4702849 26553804

[10] Lu S. , Wang J., Chitsaz F., Derbyshire M.K., Geer R.C., Gonzales N.R., Gwadz M., Hurwitz D.I., Marchler G.H., Song J.S. et al. CDD/SPARCLE: the conserved domain database in 2020. Nucleic Acids Res. 2020; 48:D265–D268. 31777944 10.1093/nar/gkz991 PMC6943070

[11] UniProt Consortium UniProt: the Universal Protein Knowledgebase in 2023. Nucleic Acids Res. 2023; 51:D523–D531. 36408920 10.1093/nar/gkac1052 PMC9825514

[12] Robinson J.T. , Thorvaldsdottir H., Turner D., Mesirov J.P. igv.js: an embeddable JavaScript implementation of the Integrative Genomics Viewer (IGV). Bioinformatics. 2023; 39:btac830. 36562559 10.1093/bioinformatics/btac830 PMC9825295

[13] Mistry J. , Chuguransky S., Williams L., Qureshi M., Salazar G.A., Sonnhammer E.L.L., Tosatto S.C.E., Paladin L., Raj S., Richardson L.J. et al. Pfam: the protein families database in 2021. Nucleic Acids Res. 2021; 49:D412–D419. 33125078 10.1093/nar/gkaa913 PMC7779014

[14] wwPDB consortium Protein Data Bank: the single global archive for 3D macromolecular structure data. Nucleic Acids Res. 2019; 47:D520–D528. 30357364 10.1093/nar/gky949 PMC6324056

[15] Jumper J. , Evans R., Pritzel A., Green T., Figurnov M., Ronneberger O., Tunyasuvunakool K., Bates R., Žídek A., Potapenko A. et al. Highly accurate protein structure prediction with AlphaFold. Nature. 2021; 596:583–589. 34265844 10.1038/s41586-021-03819-2 PMC8371605

[16] Rego N. , Koes D. 3Dmol.js: molecular visualization with WebGL. Bioinformatics. 2015; 31:1322–1324. 25505090 10.1093/bioinformatics/btu829 PMC4393526

[17] Boratyn G.M. , Schäffer A.A., Agarwala R., Altschul S.F., Lipman D.J., Madden T.L. Domain enhanced lookup time accelerated BLAST. Biol. Direct. 2012; 7:12. 22510480 10.1186/1745-6150-7-12 PMC3438057

[18] Gasiunas G. , Young J.K., Karvelis T., Kazlauskas D., Urbaitis T., Jasnauskaite M., Grusyte M.M., Paulraj S., Wang P.-H., Hou Z. et al. A catalogue of biochemically diverse CRISPR-Cas9 orthologs. Nat. Commun. 2020; 11:5512. 33139742 10.1038/s41467-020-19344-1 PMC7606464

[19] Pausch P. , Al-Shayeb B., Bisom-Rapp E., Tsuchida C.A., Li Z., Cress B.F., Knott G.J., Jacobsen S.E., Banfield J.F., Doudna J.A. CRISPR-CasΦ from huge phages is a hypercompact genome editor. Science. 2020; 369:333–337. 32675376 10.1126/science.abb1400 PMC8207990

[20] Al-Shayeb B. , Skopintsev P., Soczek K.M., Stahl E.C., Li Z., Groover E., Smock D., Eggers A.R., Pausch P., Cress B.F. et al. Diverse virus-encoded CRISPR-Cas systems include streamlined genome editors. Cell. 2022; 185:4574–4586. 36423580 10.1016/j.cell.2022.10.020

[21] Adler B.A. , Hessler T., Cress B.F., Lahiri A., Mutalik V.K., Barrangou R., Banfield J., Doudna J.A. Broad-spectrum CRISPR-Cas13a enables efficient phage genome editing. Nat. Microbiol. 2022; 7:1967–1979. 36316451 10.1038/s41564-022-01258-x PMC9712115

[22] Edgar R.C. MUSCLE: multiple sequence alignment with high accuracy and high throughput. Nucleic Acids Res. 2004; 32:1792–1797. 15034147 10.1093/nar/gkh340 PMC390337

[23] Nguyen L.-T. , Schmidt H.A., von Haeseler A., Minh B.Q. IQ-TREE: a fast and effective stochastic algorithm for estimating maximum-likelihood phylogenies. Mol. Biol. Evol. 2015; 32:268–274. 25371430 10.1093/molbev/msu300 PMC4271533

[24] Letunic I. , Bork P. Interactive Tree Of Life (iTOL) v5: an online tool for phylogenetic tree display and annotation. Nucleic Acids Res. 2021; 49:W293–W296. 33885785 10.1093/nar/gkab301 PMC8265157

[25] Goldberg G.W. , Jiang W., Bikard D., Marraffini L.A. Conditional tolerance of temperate phages via transcription-dependent CRISPR-Cas targeting. Nature. 2014; 514:633–637. 25174707 10.1038/nature13637 PMC4214910

[26] Kazlauskiene M. , Kostiuk G., Venclovas Č., Tamulaitis G., Siksnys V. A cyclic oligonucleotide signaling pathway in type III CRISPR-Cas systems. Science. 2017; 357:605–609. 28663439 10.1126/science.aao0100

[27] Niewoehner O. , Garcia-Doval C., Rostøl J.T., Berk C., Schwede F., Bigler L., Hall J., Marraffini L.A., Jinek M. Type III CRISPR-Cas systems produce cyclic oligoadenylate second messengers. Nature. 2017; 548:543–548. 28722012 10.1038/nature23467

[28] Aliaga Goltsman D.S. , Alexander L.M., Lin J.-L., Fregoso Ocampo R., Freeman B., Lamothe R.C., Perez Rivas A., Temoche-Diaz M.M., Chadha S., Nordenfelt N. et al. Compact Cas9d and HEARO enzymes for genome editing discovered from uncultivated microbes. Nat. Commun. 2022; 13:7602. 36522342 10.1038/s41467-022-35257-7 PMC9755519

[29] Urbaitis T. , Gasiunas G., Young J.K., Hou Z., Paulraj S., Godliauskaite E., Juskeviciene M.M., Stitilyte M., Jasnauskaite M., Mabuchi M. et al. A new family of CRISPR-type V nucleases with C-rich PAM recognition. EMBO Rep. 2022; 23:e55481. 36268581 10.15252/embr.202255481 PMC9724661

[30] Sun A. , Li C.-P., Chen Z., Zhang S., Li D.-Y., Yang Y., Li L.-Q., Zhao Y., Wang K., Li Z. et al. The compact Casπ (Cas12l) ‘bracelet’ provides a unique structural platform for DNA manipulation. Cell Res. 2023; 33:229–244. 36650285 10.1038/s41422-022-00771-2 PMC9977741

[31] Wu W.Y. , Mohanraju P., Liao C., Adiego-Pérez B., Creutzburg S.C.A., Makarova K.S., Keessen K., Lindeboom T.A., Khan T.S., Prinsen S. et al. The miniature CRISPR-Cas12m effector binds DNA to block transcription. Mol. Cell. 2022; 82:4487–4502. 36427491 10.1016/j.molcel.2022.11.003

[32] Chen W. , Ma J., Wu Z., Wang Z., Zhang H., Fu W., Pan D., Shi J., Ji Q. Cas12n nucleases, early evolutionary intermediates of type V CRISPR, comprise a distinct family of miniature genome editors. Mol. Cell. 2023; 83:2768–2780. 37402371 10.1016/j.molcel.2023.06.014

[33] Bravo J.P.K. , Hallmark T., Naegle B., Beisel C.L., Jackson R.N., Taylor D.W. RNA targeting unleashes indiscriminate nuclease activity of CRISPR–Cas12a2. Nature. 2023; 613:582–587. 36599980 10.1038/s41586-022-05560-w PMC9849127

[34] Dmytrenko O. , Neumann G.C., Hallmark T., Keiser D.J., Crowley V.M., Vialetto E., Mougiakos I., Wandera K.G., Domgaard H., Weber J. et al. Cas12a2 elicits abortive infection through RNA-triggered destruction of dsDNA. Nature. 2023; 613:588–594. 36599979 10.1038/s41586-022-05559-3 PMC9811890

