# Correction to ‘CasPEDIA Database: a functional classification system for class 2 CRISPR-Cas enzymes’

**DOI:** 10.1093/nar/gkad1228

**Published:** 2023-12-18

*Nucleic Acids Research*, 2023; gkad890, https://doi.org/10.1093/nar/gkad890

In the originally published version of this manuscript, the co-author's name was misspelled as ‘Michaela F. Maxwell’ and should be corrected to ‘Micaela F. Maxwell.’

Additionally, Benjamin A. Adler and Marena I. Trinidad are co-first authors with equal contributions. This has been further clarified in the author footnotes.

This error has been corrected online.

